# Socioeconomic and Health-Related Determinants of Eating Habits in Polish Caucasian Older Population—The Nationwide PolSenior2 Study Results

**DOI:** 10.3390/nu17101640

**Published:** 2025-05-11

**Authors:** Hanna Kujawska-Danecka, Jolanta A. Dardzińska, Małgorzata Mossakowska, Monika Puzianowska-Kuźnicka, Aleksandra Kaluźniak-Szymanowska, Sylwia Małgorzewicz, Edyta Wernio, Jerzy Chudek

**Affiliations:** 1 Department of Rheumatology, Clinical Immunology, Geriatrics and Internal Medicine, Medical University of Gdansk, 3a Skłodowskiej-Curie Str., 80-210 Gdansk, Poland; 2 Department of Clinical Nutrition, Medical University of Gdansk, 3a Skłodowskiej-Curie Str., 80-210 Gdansk, Poland; jolanta.dardzinska@gumed.edu.pl (J.A.D.); sylwia.malgorzewicz@gumed.edu.pl (S.M.); edyta.wernio@gumed.edu.pl (E.W.); 3 International Institute of Molecular and Cell Biology, 4 Ks. Trojdena Str., 02-109 Warsaw, Poland; mmossakowska@iimcb.gov.pl; 4 Department of Human Epigenetics, Mossakowski Medical Research Institute, PAS, 5 Pawinskiego Str., 02-106 Warsaw, Poland; mpuzianowska@imdik.pan.pl; 5 Department of Geriatrics and Gerontology, Centre of Postgraduate Medical Education, 99/103 Marymoncka Str., 01-813 Warsaw, Poland; 6 Geriatric Unit, Department of Palliative Medicine, Poznan University of Medical Sciences, 70 Bukowska Str., 61-245 Poznan, Poland; akaluzniak-szymanowska@ump.edu.pl; 7 Department of Internal Diseases and Oncological Chemotherapy, Medical University of Silesia in Katowice, 8 Reymonta Str., 40-029 Katowice, Poland

**Keywords:** eating habits, older persons, healthy diet, nutritional status, healthy aging

## Abstract

Background/Objectives: Adherence to a healthy diet may increase the chance of healthy aging. This study’s objective was to evaluate the nutritional quality of the diet and socioeconomic and health-related correlations of adherence to a healthy diet in older individuals. Methods: This analysis was part of the PolSenior2 project, which comprised 5987 respondents aged ≥60 years, representatives of the community-dwelling Polish population. Eating habits were categorized according to the Senior Healthy Diet Index (SHDI), with a score between 0 and 100 points, based on the 42-item food frequency questionnaire filled out by participants. Higher adherence to the SHDI was defined as fulfilling at least five components. Results: The mean SHDI score was significantly higher in women, 58.5 ± 11.7, compared to men (55.8 ± 11.8); *p* < 0.001. Only 0.7% of respondents were fully adherent to dietary recommendations (fulfilling at least eight of ten SHDI components). In univariate analysis, a lower prevalence of typical geriatric problems (functional impairment, dementia, depression, falls, frailty, visual impairment, lack of functional dentition) and an additional occurrence of diabetes, hypertension, and heart failure in men were significantly correlated with higher compliance with SHDI recommendations. According to multivariate regression analysis, female sex, higher education level, regular physical activity, functional dentition, diabetes, and the absence of depression or dementia were factors most strongly associated with better adherence to a healthy diet. Conclusions: Full adherence to dietary recommendations in Polish seniors is rare. Aging-related diseases correlate in various ways with better eating habits. Especially, men are more likely to choose a healthy diet when signs of deterioration appear.

## 1. Introduction

As the population ages, it becomes increasingly urgent to find solutions to the economic, social, and health challenges faced by older people. A key determinant of the aging phenotype appears to be the diet and nutritional status of older individuals [1,2,3].

Age-related changes, such as a premature sensation of satiety, impaired taste and smell perception, gastrointestinal pathologies, or functional disability, determine the diet of the elderly, which may be improper and may deteriorate their nutritional status [4,5]. A number of studies have demonstrated that both quantitative and qualitative malnutrition are associated with a poorer general prognosis and an increased risk of multimorbidity, disability, and mortality. This dynamic perpetuates a self-reinforcing cycle, known as a “vicious circle” mechanism. Furthermore, the diet of older people can be significantly influenced by socioeconomic factors, including, but not limited to, a poor financial situation, feelings of loneliness, and social isolation [4]. Consequently, it can be concluded that evaluating the nutritional quality of the diet and identifying the underlying factors that contribute to dietary decisions among the geriatric population may serve as an effective modifiable correlate of chronic diseases, characteristic geriatric issues, and the overall prognosis of the individual.

The evaluation of diet quality has recently been a subject of increasing debate [1,3,4,5]. While numerous data addressing the dietary intake of the older population have been published, these tend to focus predominantly on specific nutrients (e.g., protein) as opposed to considering the diet in its entirety. A paucity of consensus and a lack of a gold standard persist in the global literature concerning the assessment of diet quality [4,5]. The Mediterranean Diet Score [6], the Healthy Eating Index [7], and the Elderly Dietary Index [8], although well known, have only been validated in the Mediterranean basin, the United States, and Greece, respectively, while a paucity of validated tools exists in Central Europe.

It is also important to note that the diet quality indices that have been used until now have limitations in their application to older people. This issue was addressed by Dorrington et al. [5], who sought to develop three variants of the Diet Quality Index-65 (NFDQI-65, NFDQI-65+PA, FDQI-65) that take into account the intake of specific food types, nutrients, and macronutrients, as well as physical activity. The authors of this study noted that the proposed DQI components focus on the essential nutritional needs of older people, such as protein, calcium, vitamin B12, vitamin D, folic acid, dietary fiber, adequate fatty acid ratios, fluids, and limiting the intake of simple sugars and alcohol [5]. In addition, the study by Dorrington et al. enabled an examination of British senior citizens’ dietary habits, which are defined as collective, and repetitive behaviors, which lead people to select, consume, and use certain foods or diets, in response to social and cultural influences. It is worth noting that knowledge of dietary habits allows for the development of individual recommendations for patients and educational strategies for the population [9,10,11]. In turn, such education can have significant implications for improving public health, given the negative consequences of poor nutritional status on the aging trajectory.

To the best of our knowledge, there are only a few population-based studies in the literature that analyzed the dietary habits of older people in the context of various health and social factors [5,8,12]. The PolSenior2 (PS2) project created a valuable opportunity to conduct such large-scale analyses in the age-advanced Polish Caucasian population. The objective of the current analysis was to evaluate the nutritional quality of the diet in older individuals using the methodology proposed by Dorrington et al. [5]. Additionally, the study examined the influence of various factors, including socioeconomic, health-related, and age-related determinants, on the adherence to a healthy diet. It seems particularly important to identify socioeconomic factors, as they have a significant impact on dietary habits, and knowledge of them can influence social policy and enable more effective interventions in the area of social support for older people [10,13,14]. Furthermore, the identification of health-related factors, properly implemented in the field of public health, provides an opportunity to implement effective mechanisms to support successful aging through the formation of healthy eating habits. 

## 2. Materials and Methods

### 2.1. Study Protocol and Participants

The PolSenior2 survey comprised 5987 respondents aged 60 years and above, recruited as representatives of the community-dwelling Polish population. A detailed description of the sampling procedure, the study flowchart, and the protocol was published previously [15].

This study aimed to precisely characterize the health and socioeconomic status of age-advanced adults in Poland and consisted of three paper-version questionnaires, medical examination, and blood and urine collection conducted at respondents’ homes. The interviewers were nurses who underwent special training for the project. In the socioeconomic part, the respondents were asked, among others, about personal and family situation, economic status, living conditions, level of physical activity, and need for everyday help. The questionnaires also included a battery of geriatric tests and scales.

The medical part of the questionnaire consisted of general and detailed questions about past and present health status. Blood pressure and anthropometric measurements, including weight and height and waist and calf circumference, were taken. Subjects were weighed to the nearest 0.1 kg in light indoor clothing with bare feet or stockings. Using a stadiometer, height was measured without shoes and recorded to the nearest 0.5 cm. Body mass index (BMI) was calculated as the ratio of weight (kilograms) to the square of height (meters). Blood and urine sample collection was performed, and a variety of laboratory analyses were completed to assess health status. Functional status was assessed using Lawton’s Scale for Instrumental Activities of Daily Living (IADL) [16]. Cognitive functions were evaluated using the Mini Mental State Examination (MMSE) with the Mungas correction, and mood deterioration using the Geriatric Depression Scale—short version (15 items) [17,18]. 

The full version of the Mini Nutritional Assessment questionnaire (MNA) was used to diagnose malnutrition risk or malnutrition [19]. Additional information was collected regarding the quantity and quality of meals, fluid intake, tobacco smoking, and frequency of alcohol consumption. Participants, under direct supervision by a nurse, also completed the 42-item food frequency questionnaire (FFQ). In this questionnaire, they were asked to indicate how many times a day, week, or month they consumed particular product groups. As a result, the frequency of food consumption was expressed as a time/day (after converting the weekly and monthly frequency, if provided). The 42-item food frequency questionnaire used in our study is a simplified and modified version of the 62-item FFQ-6 tool, which was validated for the Polish population [20]. Some simplifications and shortening of the content were made to the original questionnaire due to the nature of the study group (seniors) and the study itself, in which diet was only part of a broad analysis. The modification of the FFQ also allows for comparison with the dietary data collected in 2008–2009 as part of the first edition of the PolSenior Study [21].

The data collection process was described in the paper presenting the PolSenior 2 study design [15] and mainly involved the conducting of questionnaires in respondents’ homes, the measurement of anthropometric data, the performance of geriatric scales and tests, and the collection of biological material for subsequent laboratory analysis.

### 2.2. Evaluation of Dietary Habits

The Senior Healthy Diet Index (SHDI) was used to describe the dietary pattern and adherence to healthy diet recommendations of the PS2 participants. This index is based on the Diet Quality Index for Older Adults (DQI-65), which was developed and used to assess adherence to dietary recommendations in the UK National Diet and Nutrition Survey [5].

In this study, the SHDI included ten components aligned with the nutritional recommendations for Polish older adults [22]. Each component could score a maximum of 10 points, leading to a total possible SHDI score of 100 points. A score of 10 was assigned for full adherence to the following recommendations:

1. Vegetables: daily frequency of consumption ≥ 3;
2. Fruit: daily frequency ≥ 2;
3. Protein (meat, dairy, eggs, fish): daily frequency ≥ 3;
4. Fish: weekly frequency ≥ 1;
5. Low-fat dairy: daily frequency ≥ 3;
6. Wholegrain carbohydrates: daily frequency ≥ 3;
7. Free sugars: daily frequency 1;
8. Fats: 100% unsaturated; no saturated fats consumed;
9. Fluids: ≥1500 mL per day;
10. Alcohol: weekly frequency ≤ 1.

Components number 7 (free sugars) and number 10 (alcohol) were scored as either 10 pts for adherence or 0 points for non-adherence. For the remaining eight components, scores ranged between 0 and 10 pts and were proportionally assigned based on the frequency of consumption, with 0 pts for non-adherence and 10 pts for full adherence. For instance, if the recommendation for vegetable consumption was ≥3 times/day, a respondent with a frequency of daily consumption 2 times/day would receive 6.7 pts. As for fats, a respondent who consumed unsaturated and saturated fats with equal daily frequency was assigned 5 pts, while a respondent who consumed only saturated fats received 0 pts.

Thus, the minimum possible total SHDI score was 0, and the closer the total score was to 100, the higher the diet quality.

The respondents were divided into two groups according to the number of components fulfilled. Fulfillment of a specific component was defined as reaching a minimum of 8 out of 10 pts. The only exceptions to this were components number 7 and 10, for which the fulfillment score was defined as 10 pts. Compliance with the 8 or more components of the Senior Healthy Diet Index was defined as full SHDI adherence. Respondents who declared fulfilling at least five SHDI components were defined as the group with higher adherence to the SHDI. In contrast, the remaining respondents formed the group with lower adherence (0–4 components).

### 2.3. Data Analysis and Covariates

For this analysis, the participants were divided into two age groups: 3807 subjects aged 60–79 (67.9% of the analyzed group) and 1797 subjects aged 80 and more (32.1%).

Apart from age and sex, the analysis included socioeconomic factors such as education (2 categories: primary or less/vocational; secondary/post-secondary/higher), place of residence (rural; urban), self-assessment of the economic situation, divided into three categories (can afford everything without saving money; living modestly to be able to afford daily expenses; money is only enough for the cheapest food or even less), type of work (blue collar/farmer; white collar), living situation (living alone; living with someone), and marital status (single—divorced, widowed, never married; involved in relationship).

Health status covariates included level of functional abilities according to the IADL (highly independent—24 pts; partially/fully dependent in advanced daily activities—23 and fewer pts), depression (6 or more points on the Geriatric Depression Scale), dementia (23 or less points on the Mini Mental State Examination), MCI (mild cognitive impairment—26–24 points in the MMSE), falls (at least one fall in the past year), vision impairment (respondents who cannot watch television or use Snellen charts—respondents who were reading lines 1–4 from the incorrect distance or reading verses 5–8 from any distance, or cannot reading any vers), constipation (stool less than once every two days), dentition status (functional dentition—min. of 20 teeth; partial edentulism—1–19 teeth; complete edentulism) [23], use of dentures, presence of chronic comorbidities (hypertension, heart failure, prediabetes and diabetes, hypercholesterolemia, stroke in a history), and smoking status (current smoker; past smokers—defined as persons who stopped smoking at least a year ago; non-smokers—persons who have never smoked). For the purposes of our analysis, we have intentionally chosen to focus on chronic diseases that are particularly associated with metabolic disorders and may result from an adherence to unhealthy nutritional habits.

Frailty syndrome was defined according to the Fried phenotype [24] with the presence of a min. of 3 out of 5 criteria (unintentional weight loss, slow gait speed, low level of physical activity, decreased grip strength, subjective feeling of exhaustion). Pre-frail status was defined as meeting 1 or 2 criteria. Robust respondents were those without even one criterion for frailty according to the Fried phenotype.

According to the WHO cut-offs, participants were categorized into 2 groups: respondents with normal BMI or overweight ≤29.9 kg/m^2^ vs. those with obesity (BMI ≥ 30 kg/m^2^) [25]. 

Nutritional status according to the MNA was classified into 2 categories: malnutrition and risk of malnutrition (≤23.5 pts) and good nutritional status (24–30 pts).

The level of physical activity was assessed in reference to the recommendations of the American College of Sports Medicine (ACSM) on cardiorespiratory exercise training [26]. In the PolSenior2 study, the ACSM recommendations were considered met if the respondents had engaged in 30 to 60 min of light- to moderate-intensity exercise 5 days per week, 20–60 min of vigorous-intensity exercise 3 days per week, or a combination of moderate and vigorous exercise.

### 2.4. Statistical Analysis

Categorical data were presented as percentages, and the continuous variables were presented using mean and standard deviation. The differences between groups were tested using the Mann–Whitney U test for quantitative data and the chi-square test for categorical variables. A multivariable logistic regression model was created to evaluate the associations between binary dependent variables and covariates. Regression coefficients were reported as odds ratios with 95% confidence intervals (95% CIs). The level of significance was set at *p* < 0.05. The data management and the statistical analyses were performed with R version 3.6.3 R (R Core Team, version 3.6.3).

## 3. Results

The food frequency questionnaire was completed by 5604 participants (93.6% of the PS2 study group); 50.7% were women and 49.3% were men. The mean SHDI score (95% CI) in the PS2 population was 57.2 (SD 11.8) and was significantly higher in women, at 58.5 (SD = 11.7), compared to men, at 55.8 (SD = 11.8); *p* < 0.001.

The values of the SHDI component scores for the entire PS2 population, as well as the percentage of individuals achieving the maximum score for each recommendation (i.e., fulfilling the recommendation), are presented in Table 1.

As shown in Table 1, respondents most frequently reported restricting alcohol consumption to once per week or less and consuming a sufficient amount of fluids. In contrast, the least commonly met components of the SHDI were adequate intake of low-fat dairy products and wholegrain carbohydrates.

**Figure 1:**
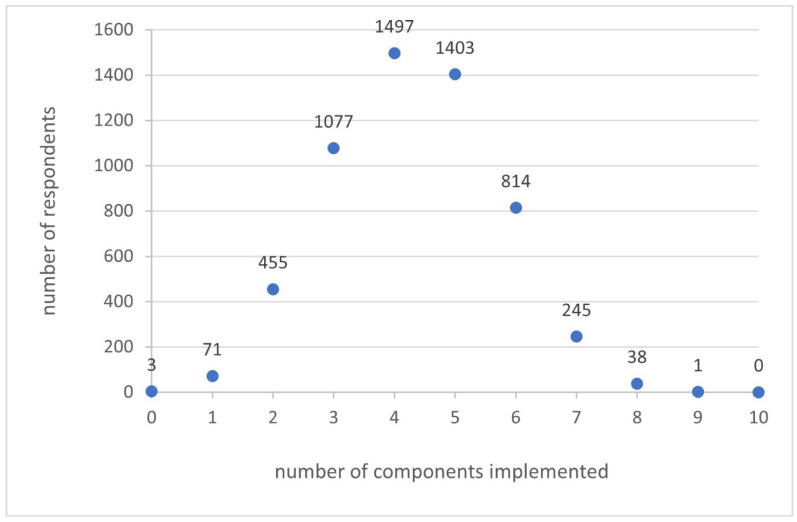
The number of respondents who implemented a particular number of components.

Full adherence to dietary recommendations (defined as a score of 8 or more in at least eight out of ten components of the SHDI) was observed in only 0.7% of respondents. The distribution of the number of fulfilled components (i.e., a score of 8 or more) in the study population is presented in Figure 1.

Table 2 and Table 3 present the characteristics of the study group according to the degree of compliance with the SHDI recommendations. A higher proportion of women in the entire study group, as well as individuals of both sexes aged 60–79, presented higher adherence to the healthy diet. Additionally, respondents residing in urban areas, individuals in stable relationships, and those with higher levels of education (high school or higher) and white collar workers were more likely to adhere to the SHDI recommendations. Similar relationships were observed when analyzing these factors among men and women separately. However, the association between living in a stable relationship and the degree of adherence to the SHDI was not significant for men (Table 4). In the higher adherence group, a larger proportion of respondents reported having sufficient financial resources to purchase all the necessary products without the necessity to make special savings, although this difference did not reach statistical significance when analyzed separately for men and women. The analysis revealed that living alone did not have a significant impact on the fulfillment of recommendations within the whole study group or separately among men and women.

Significant correlations were identified between higher compliance with SHDI recommendations and a lower prevalence of typical geriatric problems, including functional impairment, dementia, depression, falls, frailty syndrome, visual impairment, and constipation (Table 3). These correlations remained consistent in women, while no significant differences were observed among men for certain geriatric problems, such as frailty syndrome, visual impairment, and constipation (Table 4).

When both sexes were analyzed together, participants adhering to the SHDI more frequently had functional dentition.

Individuals demonstrating better adherence to the SHDI were less likely to present with malnutrition or its risk; however, they were more likely to suffer from obesity (in the whole group and men; for women, the difference was not significant).

Among individuals suffering from chronic diseases, such as diabetes, hypertension, hypercholesterolemia, heart failure, or having a history of stroke, only diabetic participants showed significantly higher adherence to the SHDI (Table 3). This association was more pronounced among men. Additionally, hypertension and heart failure were clinical conditions associated with a higher adherence to the principles of a healthy diet in men. Furthermore, regular physical activity was reported more frequently among individuals who demonstrated a higher completion of the SHDI components, a finding that was consistent across both male and female subgroups. In contrast, cigarette smoking was observed to be less prevalent among those who demonstrated a higher commitment to the SHDI components, particularly among men (Table 4).

Multivariate regression analysis (Table 5) revealed several factors significantly associated with a higher adherence to healthy dietary recommendations. These factors were female sex, higher education level, the absence of depression and dementia, functional dentition, regular physical activity, and diabetes. Conversely, the presence of a prediabetic state was found to be negatively correlated with adherence to the SHDI.

In the female cohort (Table 6), multivariate regression analysis showed that most of the above-mentioned factors were found to be associated with better adherence to a healthy diet, including secondary or higher education, the absence of depression and dementia, functional dentition, and diabetes. Additionally, a significant association was identified with regular bowel movements, defined as the absence of constipation.

In males (Table 6), no association was observed between educational level and the study objective; however, a positive correlation was identified between urban place of residence and SHDI adherence. Among the other investigated factors, functional dentition and the presence of chronic diseases, i.e., diabetes, hypertension, and heart failure, demonstrated a significant association with healthier eating habits. Additionally, regular physical activity and non-smoking status were associated with SHDI compliance. Conversely, prediabetic status was negatively correlated with adherence to healthy eating practices.

## 4. Discussion

Our study revealed that adherence to dietary recommendations was uncommon. However, adherence to these guidelines was remarkably low, with less than 1% of respondents reporting compliance. It has been demonstrated that a higher level of adherence to dietary recommendations is associated with a more advanced level of education. It is interesting to note that comorbidities such as diabetes, malnutrition, and heart failure in men are also associated with a higher level of adherence to dietary recommendations. In contrast, subjects affected by depression and dementia often demonstrate poor adherence to treatment. Therefore, inadequate adherence has the potential to pose considerable challenges for healthcare systems in aging populations. There is an urgent need to identify actions that can enhance older adults’ quality of life and functional capacity [1,27,28]. It is now indisputable that dietary habits play a crucial role in determining the risk of developing chronic diseases. This relationship is well documented for cardiovascular diseases [29,30] and cancers [31,32], two conditions that are the leading causes of morbidity among older individuals. Poor nutrition can also lead to malnutrition, the most common consequence of which is an increased incidence of infectious diseases [33,34], with the use of broad-spectrum antibiotics and frequent hospitalization. Malnutrition, often unrecognized as a disease, even by healthcare specialists, and, therefore, frequently undiagnosed, can also contribute to the development of common geriatric syndromes, such as frailty, sarcopenia, an increased risk of falls [35,36], and exacerbate preexisting depression and dementia symptoms [37,38].

Understanding the eating habits of large, representative groups of community-dwelling older people and analyzing factors that influence adherence to or deviance from healthy dietary recommendations is, therefore, crucial to developing healthy aging strategies [39]. An excellent opportunity to conduct this type of research was our nationwide PolSenior2 study that assessed dietary habits based on the frequency of consumption of selected food groups and enabled the calculation of the SHDI, specific for older adults according to Dorrington et al., which has been validated in the UK National Diet and Nutrition Survey [5].

Our results obtained in the PS2 study appear to be closely aligned with those reported by these authors. No more than one-third of Polish seniors meet the criteria for adequate vegetable and fruit intake, with the results slightly worse than those observed in UK older adults. Polish older adults, however, were slightly more likely to meet the recommended frequency of protein intake. On the other hand, the very low frequency of dairy and wholegrain consumption is concerning both in our and the British old population. Moreover, unfortunately, as many as two-thirds of participants in both populations consume simple sugar-containing products more than once a day.

Interestingly, most likely culturally driven differences were observed in the frequency of fatty fish consumption and the choice of dietary fat sources (unsaturated vs. saturated fats). Polish seniors were significantly more likely to meet the criterion of consuming fatty fish at least once a week, which may be attributed to the availability and popularity of herring. However, only 1% of our respondents reported exclusive consumption of unsaturated fats, compared to 22% of those studied by Dorrington et al. [5]. This likely reflects a higher proportion of vegans in the more multicultural British population compared to Poland.

Therefore, the dietary habits of seniors in the PolSenior2 study are highly unsatisfactory. Full adherence to the recommended diet (defined as an individual score of 8 points or more in at least eight out of ten components) was observed in less than 1% of our respondents. The vast majority of participants consumed vegetables, fruits, wholegrain products, and protein sources, including low-fat dairy, with insufficient frequency. Instead, they showed a preference for products rich in simple sugars and animal fats. Among the ten dietary recommendations assessed, the only one followed by most respondents was limiting alcohol consumption to once a week or less. However, due to the data collection method, these findings should be interpreted with caution.

To identify factors significantly associated with higher or lower adherence to dietary recommendations, our participants were categorized into two groups: those adhering to at least five out of ten dietary recommendations and those meeting only four or fewer. Similar to findings from other studies, when analyzing socioeconomic factors [8,40,41,42,43,44], our analysis revealed that individuals with better adherence were more likely to be women, younger seniors (aged 60–79), living with a partner/in relationship, residing in more urbanized areas, having a higher level of education, being in a better economic situation, and engaging in higher levels of physical activity.

An intriguing and seemingly paradoxical finding emerged when analyzing dietary adherence concerning prediabetes and diabetes diagnosis. Individuals diagnosed with prediabetes demonstrated lower rates of adherence to dietary recommendations in comparison to those with diabetes, who exhibited an increased propensity for adherence. This association may be attributed to the phenomenon of reverse causality, which is sometimes observed in cross-sectional studies. The methodology of such studies does not allow for causal inferences, meaning that the observed dietary patterns could be both a cause and a consequence of disease diagnosis. Our findings suggest that there is a lack of effective primary dietary prevention strategies for diabetes in the Polish senior population. However, receiving a diabetes diagnosis appears to lead to improved knowledge of secondary prevention measures and, importantly, positive dietary changes. The reverse causality effect in chronic diseases has also been reported in similar studies in Brazil [40] and Lebanon [42]. Interestingly, subgroup analysis in men revealed evidence of reverse causality not only in diabetes but also in hypertension and heart failure. These findings may support the hypothesis that men, who tend to pay less attention to diet quality and generally have poorer eating habits than women, have the capacity to modify lifestyle choices and transition from unhealthy dietary patterns to more beneficial alternatives in the face of health challenges. This assumption is further supported by the observation that men with a higher adherence to dietary recommendations were also more physically active and less likely to smoke.

One aspect requiring careful interpretation is the association between obesity and higher adherence to a healthier diet observed by us, particularly in men. Several key factors must be considered. First, the interpretation of body mass index (BMI) in the geriatric population is inherently limited. Aging is associated with a gradual loss of lean body mass, which becomes particularly pronounced after the age of 80. Additionally, weight loss due to anorexia of aging and the so-called “obesity paradox”, described in this population, further complicate BMI-based assessments [45,46]. The presence of heart failure and fluid retention, which was more frequent among men, also plays a role in this context. Thus, the higher proportion of men with a BMI ≥ 30 kg/m^2^ in the group with better adherence compared to those with poorer adherence to healthy diet recommendations (35% vs. 30%) might be secondary to younger age (60–79 years) or a higher prevalence of heart failure in this subgroup. This interpretation is supported by regression analysis, in which this association did not reach statistical significance. Secondly, reverse causality cannot be ruled out as a potential explanation for these findings. Thirdly, selecting healthier food products does not necessarily imply adherence to recommendations for energy intake, which can only be more accurately assessed through 24 h dietary recall—an approach that was not feasible in our large-scale study.

In the PS2 study, adherence to healthy dietary recommendations was also related to typical geriatric problems such as dementia, depression, and functional capacity. The relationship between diet and depression has been explored for years [47], and the results of many studies now suggest that this relationship is bidirectional. Following healthy dietary recommendations (including eating vegetable fats, more fish, legumes, nuts, and vegetables, and limiting the intake of highly processed foods, red meat, and simple sugars) may have an impact on reducing the risk of depression, including older adults [48,49,50,51]. A study by Gomes et al. [52] employing methodology similar to the PS2 study (a cross-sectional study using GDS to ascertain depressive symptoms) demonstrated a relationship between a healthy diet and a lower incidence of depression; however, the authors also showed an inverse correlation, as people with depression were more likely to adhere to a lower quality diet. Other researchers have also presented findings that indicate that individuals diagnosed with depression are more prone to opt for a diet of reduced nutritional value, characterized by an elevated consumption of products rich in saturated fat, sugar, and sodium and a diminished intake of fruits and vegetables [47,53,54,55]. In the PS2 study, depression also reduced the odds of meeting healthy eating recommendations. This dietary pattern may result in short-term alleviation of the depressive symptoms, which is referred to in the literature as the “reverse causality of diet and depression”. Additionally, depression is a recognized factor that adversely affects the nutritional status of the elderly, as reflected in the Mini Nutritional Assessment, where it is a high-scoring factor in the assessment of nutritional status, as is dementia [19].

In the present analysis, a significantly higher proportion of people with dementia was found in the group with lower adherence to a healthy diet. This relationship was also confirmed by regression analysis. Dementia has been shown to cause changes in eating habits at a number of levels. These alterations may arise from changes in appetite, olfactory perception, and gustatory sensitivity. Even at the early stages of cognitive impairment, activities related to the eating process, such as shopping, choosing products, finding them in the shop, and finally preparing a particular dish, can be affected. These difficulties may result in a preference for the purchase and consumption of ready-to-eat products, which typically exhibit a lower quality profile in comparison to a healthy diet. After this, the deterioration in memory may reduce the number of meals and disrupt the daily eating routine, leading to a preference for sweet products [56]. The extant literature highlights the potential of eating disorders, and weight loss in particular, as a prodromal sign of Alzheimer’s disease (AD), the most prevalent form of dementia. These symptoms may emerge before the manifestation of the typical clinical signs associated with cognitive impairment [57]. In our study, however, regression analysis did not confirm such a relationship for people with mild cognitive impairment (MCI). Concerning psychogeriatric problems, PS2 showed that for both depression and dementia, the inverse association with adherence to a healthy diet was particularly pronounced in women, while in men, it was borderline or, in the case of dementia, not relevant. The significantly different social situations of older women and men may contribute to this discrepancy. Older men are almost twice as likely as women to be in a relationship and more likely to reside with other family members. Consequently, men afflicted with depression or dementia are more likely to receive considerable assistance from their female partners or other family members, particularly with regard to activities such as shopping and meal preparation, which strongly favor adherence to healthy eating [58,59].

This perspective on disparity in health outcomes is also reflected in the survey data from the PS2, where the adherence to SHDI recommendations varies significantly by sex, functional abilities, and independence. However, in multivariate regression analysis, the relationship between better adherence to the SHDI and full self-efficiency for complex activities of daily living, as measured by the IADL scale (including shopping and meal preparation), did not reach statistical significance. Nevertheless, there were marked differences in the percentages of women and men following healthy diet recommendations according to the IADL. In women with better adherence, the proportion of functionally fit individuals was significantly higher compared to those on a poorer diet. Conversely, in men, the situation was reversed, as a significantly lower percentage of men presenting better adherence were functionally independent compared to those with lower adherence. This is due to the above-mentioned fact that men are more likely to have support with regard to meal preparation than women. Moreover, within the PS2 study, when excluding social determinants of gender roles, the entire study group with higher adherence to healthy dietary recommendations had a significantly higher percentage of independent performance in the IADL. This finding aligns with other studies that have demonstrated a parallel relationship. Kodama et al. examined the relationship between functional performance and diet among Japanese seniors, finding that those with a high intake of red meat (compared with seniors who consumed a more diversified diet rich in vegetables, fruit, and fish) were at higher risk of functional decline over a 3-year follow-up period, and this relationship was strongest among women aged 75 years and older [60]. A similar relationship was observed in the Taiwan Longitudinal Study of Aging, where high dietary diversity (including a higher content of seafood, eggs, and legumes in particular) correlated with a lower risk of functional impairment in terms of ADLs and IADL scores [61]. In addition, adherence to the MIND diet, the impact of which on IADL levels was analyzed by Agrawal et al., exhibited a favorable effect on better functional performance [44], as did the diet with a low pro-inflammatory index [62].

Oral health, understood in a broad sense (condition of the teeth, periodontal tissues, proper saliva secretion, use of dentures), has important implications for diet, and most authors emphasize that the relationship between oral health, nutritional status, and the occurrence of other typical geriatric problems is multidirectional [63,64]. In our analysis, good dental status was identified as a factor positively influencing adherence to healthy diet recommendations. However, data from the literature are inconclusive and indicate different aspects of the relationship between oral health status and diet. Iwasaki’s analysis also demonstrated that a lack of functional dentition was associated with a reduced intake of meat and vegetables [65]. In analyses of the British Regional Heart Study (BRHS) and the Health, Aging and Body Composition (HABC) Study, Kotronia et al. showed that a lack of functional dentition was associated with a reduced intake of fruit and vegetables and a high proportion of trans fats as an energy source [66]. Notwithstanding, other studies have demonstrated no association between nutrient intake and dental status and the number of teeth [67,68].

While our results offer unique contributions to the understanding of dietary habits among older adults and the factors influencing them, their interpretation requires consideration of certain limitations. First, the cross-sectional nature of our study must be taken into account. Such a study design means that the study group is not strictly selected in terms of a specific feature, and the final result may be influenced by numerous confounding factors. Second, due to the broad scope of the PS2 study, a very detailed assessment of numerous aspects of participants’ lives and health was not possible. Therefore, we did not obtain a 24 h dietary recall and, consequently, were not able to assess portion sizes, which would have allowed for calculations of total caloric intake or a precise estimation of daily nutrient intake (i.e., sodium consumption).

A major strength of the PS2 project is the large and representative sample of older adults, encompassing a wide age range, with meticulously collected data. To the best of our knowledge, this is the first study to describe the dietary patterns of such a large group of Polish Caucasian seniors and one of the largest studies of this kind in this part of Europe. Furthermore, the use of an adequate methodology allowed us to relate the data on dietary habits to a large number of other variables that accurately describe the state of health, the presence of typical geriatric problems, and social conditions.

## 5. Conclusions

In conclusion, in Polish Caucasian seniors, a high adherence to dietary recommendations is unfortunately very uncommon. Women, individuals well educated, with unaffected cognition, not depressed, physically active, and possessing functional dentition were more prone to follow these recommendations, as shown in multivariate regression analysis. A higher compliance with the SHDI correlated with a lower prevalence of geriatric syndromes. Most likely, however, the relationship between a healthier diet and a better health status is bidirectional. On the other hand, being diagnosed with some aging-related diseases also correlated with better eating habits, especially in men, which suggests an increase in interest in one’s health when it presents signs of deterioration.

Our results show low adherence to healthy diet recommendations and late implementation of them, which happens in the majority of cases after the development of serious chronic health diseases. The findings of our study also demonstrate that the key to facilitating healthy aging in our societies is the promotion of education and awareness of healthy eating habits from a young age. In light of the obesity pandemic and the associated health issues it raises, there is an urgent imperative to explore new methods of communication that can more effectively promote a healthy lifestyle.

## Figures and Tables

**Table 1:** Mean total SHDI and component scores and proportion meeting the recommendations in the entire PS2 study population (N = 5604).

	Index Component	Components for Maximum Score (i.e., 10)	Mean Score (95% CI)	Proportion Meeting Recommendation (%, 95% CI)
1.	Vegetables	daily frequency of consumption ≥ 3	6.4 (6.3–6.6)	26.7 (24.4–29)
2.	Fruit	daily frequency of consumption ≥ 2	6.0 (5.9–6.2)	32.9 (30.3–35.5)
3.	Protein	daily frequency of consumption ≥ 3	8.3 (8.2–8.4)	50.2 (48.2–52.2)
4.	Fish	weekly frequency of consumption ≥ 1	7.2 (7.1–7.4)	62.2 (59.9–64.6)
5.	Low-fat dairy	daily frequency of consumption ≥ 3	2.5 (2.4–2.6)	1.0 (0.7–1.3)
6.	Wholegrain carbohydrates	daily frequency of consumption ≥ 3	2.1 (2–2.2)	1.9 (1.4–2.5)
7.	Free sugars	daily frequency of consumption ≤ 1	- *	36.1 (33.6–38.5)
8.	Fats	100% unsaturated consumed	3.1 (2.9–3.2)	0.9 (0.5–1.4)
9.	Fluids	≥1500 mL per day	9.4 (9.3–9.5)	75.8 (73.7–77.9)
10.	Alcohol	weekly frequency of consumption ≤ 1	- *	93.6 (92.6–94.5)

* For components 7 and 10, the possible scoring was 0 or 10 points.

**Table 2:** Sociodemographic characteristics concerning adherence to the SHDI for the whole study group.

	Total (N = 5604)	Adherence to SHDI		*p*-Value
		Lower (0–4 Components) (n = 3103)	Higher (≥5 Components) (n = 2501)	
Total, %		55.4	44.6	
Women, %	50.7	48.0	54.1	<0.001
Men, %	49.3	52.0	45.9	
Age, years	74.8 ± 9.4	75.7 ± 9.7	73.6 ± 8.9	<0.001
Age category, %				
60–79 years	67.9	63.8	73.0	<0.001
80 and more years	32.1	36.2	27.0	
Living alone, %	21.1	21.5	20.6	<0.05
Marital status, %				
In a relationship	61.0	59.2	63.3	<0.01
Single (divorced, widower, never married)	39.0	40.8	36.7	
Place of residence, %				
Rural	35.2	38.1	31.7	<0.001
Urban	64.8	61.9	68.3	
Education, %				
Primary or less/basic vocational	53.0	57.3	47.8	<0.001
Secondary/post-secondary/higher	47.0	42.7	52.2	
Type of work, %				
Blue collar/farmer	63.2	66.3	59.4	<0.001
White collar	34.8	31.9	38.4	
Economic status, %				
Can afford everything without saving money	18.9	18.1	19.9	<0.05
Living modestly to be able to afford daily expenses	74.0	74.2	73.8	
Money is only enough for the cheapest food, or even less	7.1	7.7	6.3	
Current or past smoker, %	50.9	52.0	49.6	0.074
Physical activity, %				
Meets ACSM criteria	33.7	31.2	36.9	<0.001

ACSM—American College of Sports Medicine.

**Table 3:** Health status characteristics concerning adherence to the SHDI for the whole study group.

	Total (N = 5604)	Adherence to SHDI		*p*-Value
		Lower (0–4 Components) (n = 3103)	Higher (≥5 Components) (n = 2501)	
IADL, %				
Highly functional (24 pts)	34.2	61.6	71.0	<0.001
Partially disabled or disabled (≤23 pts)	65.8	38.4	29.0	
Depression, %	26.2	29.7	22.1	<0.001
Cognitive impairment, %				
Without	52.7	48.2	58.2	<0.001
MCI	22.7	23.0	22.5	
Dementia	24.6	28.8	19.3	
Falls, %	19.0	20.4	17.3	<0.01
Frailty, %	22.6	21.5	19.5	<0.001
Vision impairment, %	46.4	48.1	44.3	<0.01
Constipation, %	9.4	10.2	8.4	<0.05
Dentition, %				
Functional dentition (≥20 teeth)	15.4	13.1	18.3	<0.001
Partial edentulism (1–19 teeth)	48.2	47.9	48.7	
Complete edentulism	36.4	39.0	33.1	
Dentures, % (only in the group with <20 teeth)				
Used	81.7	80.6	83.2	<0.05
Not used	18.3	19.4	16.8	
BMI category, %				
Normal/overweight (18.5–29.9 kg/m^2^)	63.3	64.7	61.6	<0.01
Obesity (≥30 kg/m^2^)	35.8	34.2	37.8	
MNA nutritional status, %				
Good	71.7	69.9	73.9	<0.001
Risk of malnutrition or malnutrition	28.3	30.1	26.1	
Heart failure, %	21.0	20.7	21.2	0.661
Hypertension, %	77.2	76.4	78.2	0.089
Past stroke, %	9.0	9.3	8.7	0.475
Diabetes, %	25.7	23.4	28.5	<0.001
Hypercholesterolemia, %	75.8	75.2	76.5	0.265

BMI—body mass index, IADL—Instrumental Activities of Daily Living, MCI—mild cognitive impairment, MNA—Mini Nutritional Assessment.

**Table 4:** Health status and sociodemographic characteristics of women and men concerning adherence to the SHDI.

	Women Adherence to SHDI			Men Adherence to SHDI		
	Lower (0–4 Components) (n= 1488)	Higher (≥5 Components) (n = 1353)	*p*-Value	Lower (0–4 Components) (n= 1615)	Higher (≥5 Components) (n = 1148)	*p*-Value
Age, years	75.9 ± 9.7	72.9 ± 8.7	<0.001	75.5 ± 9.6	74.2 ± 9.4	<0.001
Age category, %						
60–79 years	61.8	75.1	<0.001	65.8	70.6	<0.01
80 and more years	38.2	24.9		34.2	29.4	
Living alone, %	30.5	27.8	0.121	13.2	12.1	0.417
Marital status, %						
In a relationship	39.5	49.5	<0.001	77.4	79.4	0.200
Single (divorced, widower, never married)	60.5	50.5		22.6	20.6	
Place of residence, %						
Rural	39.2	31.5	<0.001	37.0	32.0	<0.01
Urban	60.8	68.5		63.0	68.0	
Education, %						
Primary or less/basic vocational	57.1	45.5	<0.001	57.2	50.4	<0.001
Secondary/post-secondary/higher	42.9	54.5		42.5	49.6	
Type of work, %						
Blue collar/farmer	60.3	53.4	<0.01	71.7	66.4	<0.05
White collar	37.7	44.2		26.7	31.8	
Economic status, %						
Can afford everything without saving money	14.7	16.6	0.137	21.2	23.8	0.108
Living modestly to be able to afford daily expenses	75.9	75.7		72.7	71.5	
Money is only enough for the cheapest food, or even less	9.4	7.7		6.1	4.7	
IADL, %						
Highly functional (24 pts)	59.7	72.5	<0.001	36.6	30.8	<0.01
Partially disabled or disabled (23 and less pts)	40.3	27.5		63.4	69.2	
Depression, %	34.4	24.3	<0.001	25.5	19.6	<0.001
Cognitive impairment, %						
Without	45.3	58.7	<0.001	50.9	57.5	<0.001
MCI	23.2	22.4		22.7	22.6	
Dementia	31.5	18.8		26.4	19.9	
Falls, %	24.8	21.0	<0.05	16.3	12.9	<0.05
Frailty, %	27.3	17.6	<0.001	23.0	21.8	0.761
Vision impairment, %	49.8	42.5	<0.001	46.6	46.5	0.934
Constipation, %	13.1	10.6	<0.001	7.5	9.1	0.128
Dentition, %						
Functional dentition (≥20 teeth)	11.6	18.2	<0.001	14.4	18.4	<0.01
Partial edentulism (1–19 teeth)	45.5	46.5		50.2	51.3	
Complete edentulism	42.9	35.3		35.4	30.4	
Dentures (only in group with less than 20 teeth), %						
Used	86.0	88.2	0.131	75.4	77.1	0.376
Not used	14.0	11.8		24.6	22.9	
BMI category, %						
Normal/overweight 18.5–29.9 kg/m^2^)	60.0	59.1	0.102	69.1	64.6	<0.05
Obesity (≥30 kg/m^2^)	38.7	40.4		30.1	34.7	
Nutritional status—MNA, %						
Good	65.5	72.1	<0.001	74.0	76.1	0.213
Risk of malnutrition/malnutrition	34.5	27.9		26.0	23.9	
Heart failure, %	19.0	17.3	0.250	22.4	25.9	<0.05
Hypertension, %	77.5	77.3	0.856	75.3	79.5	<0.01
Past stroke, %	8.8	6.8	<0.05	9.7	11.0	0.267
Diabetes, %	21.8	24.9	0.154	24.9	32.8	<0.001
Hypercholesterolemia, %	79.4	80.0	0.705	71.4	72.4	0.553
Current or past smoker, %	33.0	35.8	0.119	69.4	65.8	<0.05
Physical activity, %						
who meet ACSM criteria	26.7	34.3	<0.001	35.3	40.0	<0.05

ACSM—American College of Sports Medicine, IADL—Instrumental Activities of Daily Living, MCI—mild cognitive impairment, MNA—Mini Nutritional Assessment, BMI—body mass index.

**Table 5:** Factors for a higher adherence to the Senior Healthy Diet Index in the multivariate regression model.

	OR *	Lower CI	Higher CI	*p*-Value
Men	0.67	0.58	0.78	<0.001
Age 60–79	1.13	0.95	1.36	0.18
In relationship	1.06	0.91	1.24	0.45
Living in an urban area	1.15	0.99	1.33	0.07
Education (secondary/higher)	1.32	1.09	1.60	<0.01
White collar	0.86	0.71	1.05	0.14
Highly independent (IADL 24 pts)	1.07	0.88	1.29	0.48
BMI 18.5–29.9 kg/m^2^	0.92	0.79	1.06	0.24
Malnutrition/ at risk of malnutrition	1.26	1.04	1.53	0.02
Depression	0.67	0.56	0.81	<0.001
Mild cognitive impairment	1.12	0.90	1.40	0.30
Dementia	0.75	0.61	0.93	0.01
No falls	1.06	0.88	1.29	0.52
Pre-frail	0.91	0.73	1.14	0.42
Robust	0.87	0.66	1.13	0.28
Good vision	0.99	0.86	1.13	0.85
No constipation	1.18	0.91	1.52	0.21
Partial edentulism	1.08	0.93	1.26	0.33
Functional dentition	1.45	1.16	1.80	<0.01
Prediabetes	0.73	0.60	0.89	<0.01
Diabetes	1.47	1.25	1.75	<0.001
Meets ACSM criteria	1.22	1.06	1.42	<0.01

* OR—Odds Ratio.

**Table 6:** Factors for a higher adherence to the Senior Healthy Diet Index in the multivariate regression model—separately for women and men.

	Women				Men			
	OR	Lower CI	Higher CI	*p*-Value	OR	Lower CI	Higher CI	*p*-Value
Age 60–79	1.25	0.96	1.64	0.10	0.99	0.78	1.26	0.93
In relationship	1.11	0.90	1.36	0.33				
Living in an urban area	1.15	0.92	1.42	0.21	1.27	1.04	1.56	0.02
Education (secondary/higher)	1.62	1.19	2.20	<0.01	1.19	0.94	1.51	0.16
White collar	0.66	0.49	0.90	<0.01	0.96	0.75	1.24	0.75
Highly independent (IADL 24 pts)	1.08	0.82	1.42	0.59	0.98	0.77	1.24	0.86
BMI 18.5–29.9 kg/m^2^	NI *				0.82	0.67	1.00	0.05
Malnutrition/at risk of malnutrition	1.49	1.14	1.96	<0.01				
Depression	0.65	0.51	0.84	<0.01	0.79	0.63	1.00	0.05
Mild cognitive impairment	1.09	0.80	1.49	0.60	1.02	0.76	1.37	0.90
Dementia	0.72	0.53	0.98	0.03	0.89	0.67	1.18	0.43
No falls	0.93	0.73	1.19	0.56	1.27	0.95	1.70	0.11
Pre-frail	1.15	0.85	1.57	0.36	NI *			
Robust	1.16	0.80	1.69	0.44	NI *			
Good vision	1.18	0.96	1.43	0.11	NI *			
No constipation	1.82	1.29	2.58	<0.001	NI *			
Partial edentulism	0.97	0.78	1.21	0.80	1.19	0.96	1.47	0.11
Functional dentition	1.40	1.02	1.93	0.04	1.36	1.01	1.82	0.04
Prediabetes	0.75	0.56	1.01	0.06	0.75	0.58	0.96	0.02
Diabetes	1.33	1.05	1.69	0.02	1.47	1.16	1.85	<0.01
Heart failure	NI *				1.30	1.04	1.61	0.02
Hypertension	NI *				1.28	1.61	1.02	0.03
Meets ACSM criteria	1.15	0.93	1.42	0.21	1.26	1.04	1.52	0.02
No smoking (present or past)	NI *				1.23	1.00	1.50	0.05

* NI—not included.

## Data Availability

The data presented in this study are available upon request from the corresponding author (the data are not publicly available due to some legal issues—the owner of the database is the Ministry of Health).
